# Supplementary material for: The Evolution and Characterization of the RNA Interference Pathways in Lophotrochozoa
Source: Genome Biol Evol. 2024 May 7;16(5):evae098. doi: 10.1093/gbe/evae098 (PMC11114477; doi:10.1093/gbe/evae098)
Supplement: evae098_Supplementary_Data [file evae098_supplementary_data.zip › Supplementary_materials.zip!/selected_miRNAs/NoGe_selected_miRNAs/mirdeep_pdf_files/NMRB01000004.1_Notospermus_geniculatus_strain_Ushimado_scaffold4,_whole_genome_shotgun_sequence_167.pdf]

Provisional ID	:	NMRB01000004.1_Notospermus_geniculatus_strain_Ushimado_scaffold4,_whole_genome_shotgun_sequence_167
Score total	:	131
Score for star read(s)	:	3.9
Score for read counts	:	126.6
Score for mfe	:	1.1
Score for randfold	:	0
Score for cons. seed	:	-0.6
Total read count	:	260
Mature read count	:	199
Loop read count	:	0
Star read count	:	61

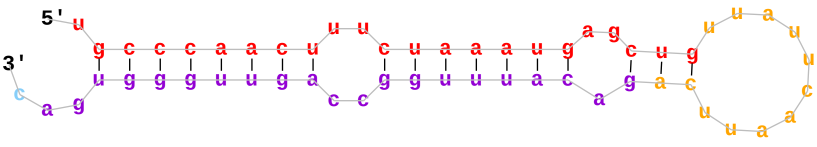

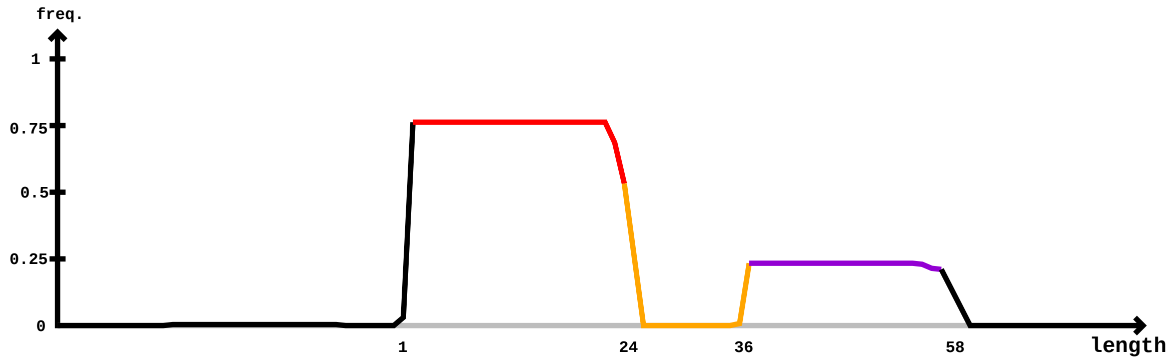


```
                                  Mature                           Star
5'- aucugaguugccaaagaacauugcuccuggauuguugcccaacuuucuaaaugagcuguuauucaauucagacauuuggccaguuggguga cuguuucagguugagcucca  -3'   obs
    aucugaguugccaaagaacauugcuccuggauuguugcccaacuuucuaaaugagcuguuauucaauucagacauuuggccaguugggugacuguuucagguugagcucca         exp
    ......(((.......)))...(((((((((..(((((((((((..(((((((..(((..........))).)))))))..)))))))))))...))))))...)))....  reads  mm  sample
    ..................................uugcccaacuuucuaaaCgagc.......................................................  1      1   Mm2
    ..................................uuAcccaacuuucuaaaugagcu......................................................  1      1   Mm2
    ..................................uugcccaacuuucuaaaugagcu......................................................  4      0   Mm2
    ..................................uuAcccaacuuucuaaaugagcug.....................................................  1      1   Mm2
    ...................................uAcccaacuuucuaaaugagc.......................................................  18     1   Mm2
    ...................................uAcccaacuuucuaaaugagcu......................................................  35     1   Mm2
    ...................................uAcccaacuuucuaaaugagcug.....................................................  136    1   Mm2
    ...................................uAcccaacuuucuaaaugagcugu....................................................  2      1   Mm2
    .....................................................................agacauuuggccaguuggguga....................  1      0   Mm2
    .....................................................................agacauuuggccUguuggguga....................  1      1   Mm2
    ......................................................................gacauuuggccaguuggg.......................  1      0   Mm2
    ......................................................................gacauuuggccaguugggug.....................  1      0   Mm2
    ......................................................................gacauuuggccaguugggugG....................  1      1   Mm2
    ......................................................................gacauuuggccaguugCguga....................  1      1   Mm2
    ......................................................................gacauuuggccaguuggguga....................  10     0   Mm2
    ......................................................................gacauuuggccaguugggugac...................  6      0   Mm2
    ......................................................................gacauuuggccaguugggugaU...................  2      1   Mm2
    ......................................................................gacauuuggccaguuggCugac...................  1      1   Mm2
    ......................................................................gacauuuggccaguugggugacu..................  1      0   Mm2

    ......................................................................Uacauuuggccaguuggguga....................  1      1   Mm1
    ......................................................................gacauuuggccaguuggguga....................  12     0   Mm1
    ......................................................................gacauuuggccaguugggugac...................  5      0   Mm1
    ......................................................................gacauuuggccaguugggugaU...................  4      1   Mm1

    ..........ccGaagaacauugcuccu...................................................................................  1      1   Mm3

    ......................................................................gacauuuggccaguugggu......................  1      0   Fm1
    ......................................................................gacauuuggccaguuggguga....................  6      0   Fm1
    ......................................................................gacauuuggccaguugggugG....................  1      1   Fm1
    ......................................................................gacauuuggccaguugggugacu..................  2      0   Fm1

    ..................................uugcccaacuuucuaaaugagc.......................................................  1      0   Fm3
```

```
                                   Mature                              Star
aucugaguugccaaagaacauugcuccuggauuguugcccaacuuucuaaaugagcuguuauucaauucagacauuuggccaguugggugacuguuucagguugagcucca
......................................................................gacauuuggccaguugggu......................	3	0	Fm3
```
